# Multi-trajectories of triglyceride-glucose index and lifestyle with Cardiovascular Disease: a cohort study

**DOI:** 10.1186/s12933-023-02076-z

**Published:** 2023-12-13

**Authors:** Hui Zhou, Xiong Ding, Yulong Lan, Shuohua Chen, Shouling Wu, Dan Wu

**Affiliations:** 1 Nursing Department, The Third Xiangya Hospital, Central South University, Changsha, China; 2 Xiangya School of Nursing, Central South University, Changsha, China; 3 School of Public Health, Wuhan University, Wuhan, China; 4 Global Heath Research Center, Duke Kunshan University, Kunshan, Jiangsu Province China; 5 Second Affiliated Hospital of Shantou University Medical College, Shantou, China; 6 Centre for Precision Health, School of Medical and Health Sciences, Edith Cowan University, Joondalup, WA Australia; 7 Department of Cardiology, Kailuan General Hospital, 57 Xinhua East Rd, Tangshan, China

**Keywords:** Cohort study, Triglyceride-glucose, Lifestyle, Multi-trajectory, Cardiovascular Disease

## Abstract

**Background:**

Previous studies using trajectory models focused on examining the longitudinal changes in triglyceride-glucose (TyG) levels and lifestyle scores separately, without exploring the joint evolution of these two factors. This study aimed to identify the multi-trajectories of TyG levels and lifestyle scores and assess their association with the risk of cardiovascular disease (CVD).

**Methods:**

The study enrolled 47,384 participants from three health surveys of the Kailuan Study. The TyG index was computed as Ln [fasting triglycerides (mg/dL) × fasting blood glucose (mg/dL)/2], and the lifestyle scores were derived from five factors, including smoking, alcohol consumption, physical activity, sedentary behaviors, and salt intake. A group-based multi-trajectory model was adopted to identify multi-trajectories of TyG levels and lifestyle scores. The association of identified multi-trajectories with incident CVD was examined using Cox proportional hazard model.

**Results:**

Five distinct multi-trajectories of TyG levels and lifestyle scores were identified. During a median follow-up period of 10.98 years, 3042 participants developed CVD events (2481 strokes, 616 myocardial infarctions, and 55 co-current stroke and myocardial infarctions). In comparison to group 3 with the lowest TyG levels and the best lifestyle scores, the highest CVD risk was observed in group 5 characterized by the highest TyG levels and moderate lifestyle scores (HR = 1.76, 95% CI: 1.50–2.05). Group 2 with higher TyG levels and the poorest lifestyle scores had a 1.45-fold (95% CI 1.26–1.66) risk of CVD, and group 1 with lower TyG levels and poorer lifestyle scores had a 1.33-fold (95% CI 1.17–1.50) risk of CVD. Group 4, with moderate TyG levels and better lifestyle scores, exhibited the lowest CVD risk (HR = 1.32, 95% CI: 1.18–1.47).

**Conclusions:**

Distinct multi-trajectories of TyG levels and lifestyle scores corresponded to differing CVD risks. The CVD risk caused by a high level TyG trajectory remained increased despite adopting healthier lifestyles. These findings underscored the significance of evaluating the combined TyG and lifestyle patterns longitudinally, and implementing early interventions to reduce CVD risk by lowering TyG levels.

**Supplementary Information:**

The online version contains supplementary material available at 10.1186/s12933-023-02076-z.

## Introduction

Cardiovascular disease (CVD) is the leading cause of morbidity and mortality worldwide [1]. Diabetes, metabolic syndrome, and obesity are known to be important, modifiable risk factors for CVD, all characterized by insulin resistance [2]. The triglyceride-glucose (TyG) index, calculated from fasting triglyceride and blood glucose levels, offers an improved and cost-effective marker for identifying insulin resistance [3, 4]. A growing number of studies have reported the predictive value of the TyG index in CVD. In the Iranian population, each standard deviation increase in the TyG index was associated with a 1.16-fold increase in the CVD risk and a 1.19-fold increase in coronary heart disease risk [5]. A linear association between the TyG index and stroke risk has been observed as well [6].

Given the dynamic nature of both CVD progression and individual TyG levels, assessing only a single time-point TyG level has limitations. Utilizing trajectory modeling enables the tracking of individual trends and recognition of diverse change patterns, and offers more precise approaches for personalized CVD prevention and intervention [7]. Few studies examined the association between changes in TyG over time and CVD incidence, and the results indicated that individuals with TyG trajectory at a high level were independently associated with an increased CVD risk, regardless of the baseline TyG index [8, 9].

However, the existing investigations did not consider the correlation of TyG with lifestyle and assessed the effect of TyG on CVD separately. Notably, the Healthy Lifestyle Promotion Program for Yaquis revealed significant short- and medium-term decreases in the Homeostasis Model Assessment of Insulin Resistance and TyG index among program completers [10]. In another randomized controlled trial, diabetes-related markers, including TyG, weight, fasting glucose, and triglyceride, also improved across all four lifestyle interventions [11]. A healthy lifestyle holds a crucial role in the context of overall well-being, with long-term benefits for CVD reduction [12, 13]. While evidence showed independent changes in TyG and lifestyle, co-evolution patterns in both remained unknown. Moreover, there was an absence of research on the impact of combined TyG and lifestyle trajectories on CVD.

Consequently, we used the Kailuan Study and employed group-based multi-trajectory modeling, a discovery-oriented statistical approach, to identify longitudinal multi-trajectories of TyG levels and lifestyle scores within the Chinese population. This approach enabled the identification of subgroups with similar changing patterns over time. We also examined the associations between the identified multi-trajectory groups and subsequent CVD risk, to further characterize risk differences between different subgroups.

## Methods

The data that support the findings of this study are available from the corresponding author upon reasonable request.

### Study population and study design

**Fig. 1:**
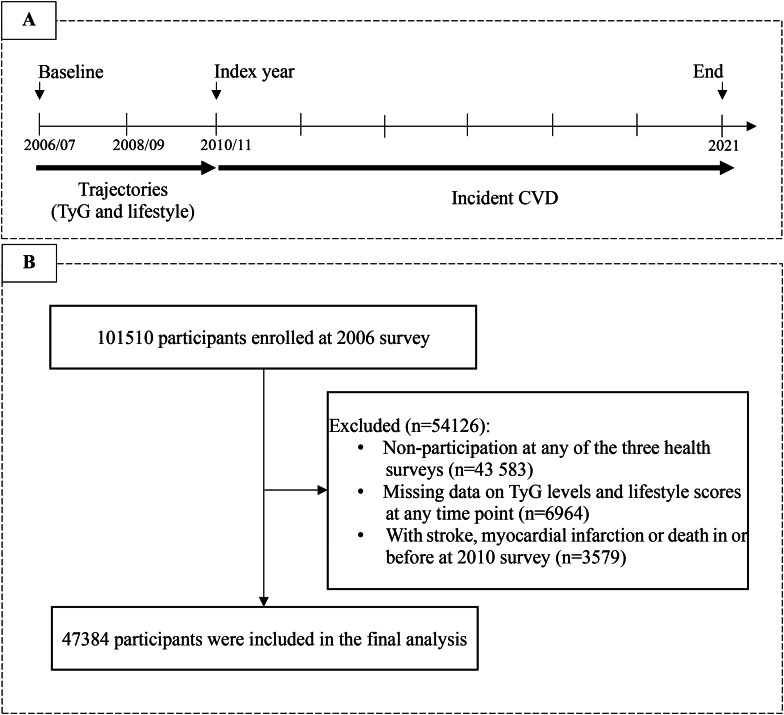
**A**) Flow chart for the selection of study participants; **B**) Study design for examine examining the association between multi-trajectories and CVD risk

Participants were recruited from the Kailuan Study, a prospective cohort conducted at Kailuan General Hospital and its 10 affiliated hospitals. The study details have been previously described [14]. Briefly, participants from the Kailuan community completed the initial survey through face-to-face interviews between June 2006 and October 2007, followed by biennial follow-ups. The study’s objectives and design were previously published and registered with the Chinese Clinical Trial Registry (Registration No.: ChiCTR-TNRC11001489). Our study had two parts: first, to identify joint developmental trajectories of TyG levels and lifestyle scores from 2006 to 2010 survey in the population; second, to predict CVD after 2010 survey. To meet the temporal requirements for multi-trajectory modeling and allow for a sufficiently long follow-up for incident events, data from health surveys conducted in 2006/07, 2008/09, and 2010/11 survey were included. Participants were excluded based on: (1) missing any of the three health surveys; (2) incomplete data on TyG levels and lifestyle scores at any time point; (3) a history of CVD in or before the third health survey. Ultimately, 47,384 participants were included in the final analytic sample (Fig. 1). Approval for this study was granted by the Ethics Committees of Kailuan General Hospital (Approval No.: 2006-5), and written informed consent was acquired from all enrolled participants.

### Definition of lifestyle scores

Trained staff employed a standard questionnaire to gather lifestyle information including smoking status, alcohol consumption, physical activity, sedentary behavior, and salt intake. Each lifestyle aspect received a score on a scale from 0 (poor) to 2 (ideal), detailed in Supplementary Table 1. Total scores ranged from 0 (worst) to 10 (best) [13].

Current smokers were defined as those who smoked daily for at least six months prior. Past smokers had quit before or during the survey. Similarly, current drinkers had consumed alcohol at least once a month in the past year, while past drinkers had quit before or during the survey. Sedentary behavior was categorized as poor (≥ 8 h/day), intermediate (4–7 h/day), or ideal (< 4 h/day). Intermediate physical activity involved ≥ 20 min per session, 1–2 times weekly, while ideal activity meant ≥ 3 times per week during leisure. Due to limited dietary data from 2006 to 2010 survey and considering the impact of salt on CVD in China, salt intake was measured by questionnaire as a proxy for diet quality, which has been validated in previous studies, with a strong association between higher perceived salt intake and lower healthy diet score [15]. Poor, intermediate, and ideal diets corresponded to salt intakes of ≥ 10 g/day, 6–9 g/day, and < 6 g/day.

### Definition of the TyG index

The TyG index was computed as Ln [fasting triglycerides (mg/dL) × fasting blood glucose (mg/dL)/2] [16]. fasting blood glucose and fasting triglycerides were measured using standard laboratory methods at Kailuan General Hospital. Blood samples (approximately 5 ml) were drawn from the cephalic vein in the morning after an overnight (> 8 h) fast. Fasting blood glucose was analyzed using the hexokinase method (< 2% coefficient of variation, upper linearity limit: 33.3 mmol/L), and serum fasting triglycerides were assessed using the enzymatic colorimetric method. Hematological parameters were analyzed with an autoanalyzer (Hitachi, Tokyo, Japan).

### Assessment of outcomes

The primary outcome of our study was the first occurrence of CVD (including stroke and myocardial infarction). The diagnosis of CVD has been previously described, employing the tenth revision of the International Classification of Diseases (ICD-10) codes for identification (MI: I21, stroke: 160–163). All participants were linked to the municipal social insurance institution and the hospital discharge register system to obtain incidence data for CVD, updated annually. This coverage included all participants of the Kailuan Study. To further identify potential CVD events, we reviewed the discharge lists from 11 hospitals between 2006 and 2021. During biennial health surveys, we gathered insights into participants’ CVD history via questionnaires. A panel of three experienced physicians independently reviewed medical records for suspected CVD cases without knowledge of the study design. The diagnosis of stroke was based on neurological signs, clinical symptoms, and neuroimaging tests, including computed tomography or magnetic resonance imaging, according to the World Health Organization criteria [17]. Myocardial infarction was diagnosed based on clinical symptoms, changes in serum concentrations of cardiac enzymes and biomarkers, and electrocardiographic results [18].

### Covariates at baseline

Data on covariates were acquired via questionnaires, basic anthropometric measurements, and laboratory assessments. The demographics collected included age, sex, marital status, and educational background. Medical history and medication usage were assessed by self-report and medical record reviews. Hypertension was defined as systolic blood pressure (SBP) ≥ 140 mmHg or diastolic blood pressure (DBP) ≥ 90 mmHg, antihypertensive drug usage, or self-reported history of physician-diagnosed hypertension. Diabetes mellitus was defined as self-reported diabetes mellitus, glucose-lowering drug usage, or fasting blood glucose ≥ 7 mmol/L. Weight, height, SBP, and DBP were all measured by trained staff. Body mass index (BMI) was computed by dividing weight in kilograms by height in meters squared [19]. The estimated glomerular filtration rate (eGFR) was calculated using the Chronic Kidney Disease Epidemiology Collaboration creatinine equation [20].

All laboratory tests, including high-density lipoprotein cholesterol (HDL-C), low-density lipoprotein cholesterol (LDL-C), high-sensitivity C-reactive protein (Hs-CRP), and serum creatinine, were performed using Hitachi 7600 auto-analyzer.

### Statistical analysis

We employed the group-based multi-trajectory modeling, using PROC TRAJ in SAS software [21], to identify jointly longitudinal changes of TyG levels and lifestyle scores within the Kailuan Study. This approach aimed to capture similar changing patterns over time in two variables [7]. Notably, when constructing the model and assigning cluster membership, both TyG levels and lifestyle scores at each visit were taken into consideration. To determine the optimal number of stable trajectories for inclusion in the multi-trajectory model and validate statistical assumptions, separate estimations were initially conducted for each dimension. Model comparison started with a single category and progressively increased the categories until model fit indicators no longer met the recommended criteria or were unable to differentiate meaningful trajectories. We evaluated fit indices including lower absolute Bayesian Information Criterion, log-likelihood ratio compared to the prior model, posterior probabilities of group allocation > 0.7, group size > 5%, and odds of correct classification > 5. Subsequently, data visualization methods were employed to evaluate data separation and model fit, following specific reporting guidelines [22].

Person-years were calculated from the date of the 2010 survey to the date of the first occurrence of CVD, death, or the end of the follow-up (31 December 2021), whichever came first. Kaplan-Meier method was utilized to estimate CVD probabilities and log-rank tests were employed to compare inter-group differences. To explore the association between multi-trajectory groups and the occurrences of CVD, stroke, and myocardial infarction, we conducted Cox proportional hazards regression for each outcome separately. Group 3, characterized by the lowest TyG levels and the best lifestyle scores, was used as the reference. Hazard ratios (HRs) and 95% confidence intervals (CIs) were reported as results. The model met the proportional assumption criteria according to Schoenfeld residuals and log-log tests. Our study comprised three models: Model 1 adjusted for age and sex. Model 2 further adjusted for marital status, education background, and BMI. Model 3 further adjusted for HDL-C, LDL-C, Hs-CRP, eGFR, hypertension, diabetes mellitus, antihypertensive, hypoglycemic, and hypolipidemic medications.

We performed stratified analyses by age, sex, diabetes mellitus and hypertriglyceridemia to assess whether the influence of TyG levels and lifestyle scores multi-trajectories on CVD varied across specific factors. We employed likelihood ratio tests to evaluate interactions between stratified variables and multi-trajectories. To test the robustness of our findings, we performed several sensitivity analyses. First, we adjusted for baseline TyG levels and lifestyle scores to explore whether the effect of multi-trajectories on CVD was independent of baseline conditions. Second, we excluded participants using medications as a separate analysis. Furthermore, we excluded participants with inflammation condition (Hs-CRP ≥ 10 mg/L). To mitigate reverse causality, we conducted a lagged analysis excluding CVD events within the initial 2 years of follow-up. Lastly, we applied a Fine-Gray competing risk model, treating non-CVD deaths as competing risk events.

All analyses were conducted using SAS version 9.4 (SAS Institute Inc., Cary, NC). A 2-sided *P* < 0.05 was considered statistically significant.

## Results

### Baseline characteristics

**Fig. 2:**
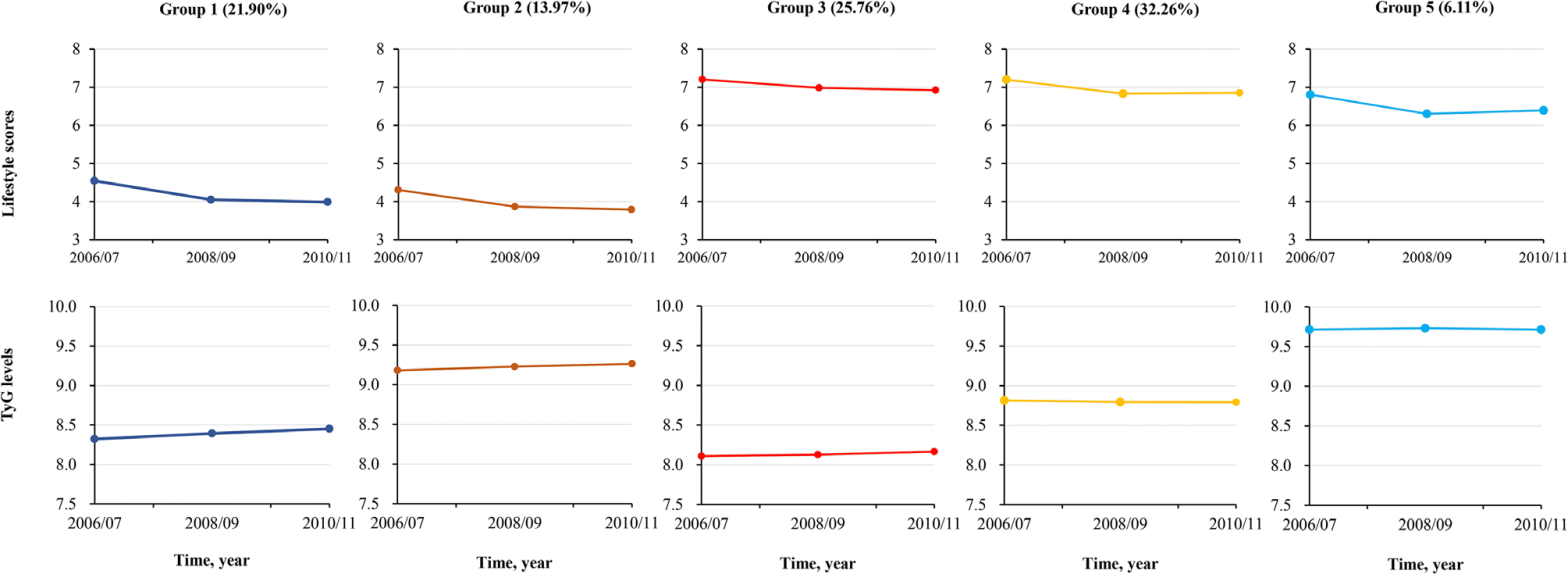
Multi-trajectory groups of TyG levels and lifestyle scores using group-based multi-trajectory modeling

A total of 47,384 participants were enrolled in the current study, with a mean age of 52.42 ± 11.77 years. We identified five distinct multi-trajectories from all investigated models. Trajectories were labeled as group 1 (lower TyG levels and poorer lifestyle scores, n = 10,469, 21.90%), group 2 (higher TyG levels and the poorest lifestyle scores, n = 6559, 13.97%), group 3 (the lowest TyG levels and the best lifestyle scores, n = 11,974, 25.76%), group 4 (moderate TyG levels and better lifestyle scores, n = 15,621, 32.26%), and group 5 (the highest TyG levels and moderate lifestyle scores, n = 2761, 6.11%) detailed in Fig. 2. The average posterior probability of the five multi-trajectories was 0.84 (Supplementary Table 2). Table 1 presented the basic characteristics of participants grouped by multi-trajectories of TyG levels and lifestyle scores. Compared with other groups, participants in group 5 were more likely to be older; men; have hypertension, diabetes mellitus, and dyslipidemia; more likely to take antihypertensive, hypoglycemic, and hypolipidemic medications; and have a higher level of BMI, SBP, DBP, LDL-C, and Hs-CRP.

**Table 1:** Basic characteristics of participants

Characteristics	Total	Group 1	Group 2	Group 3	Group 4	Group 5
N	47,384	10,469	6559	11,974	15,621	2761
Age, year	52.42 ± 11.77	49.66 ± 11.17	48.59 ± 9.63	53.28 ± 12.76	54.87 ± 11.61	54.36 ± 10.55
Sex						
Female	11,375 (24.01)	197 (1.88)	48 (0.73)	5259 (43.92)	5202 (33.30)	669 (24.23)
Male	36,009 (75.99)	10,272 (98.12)	6511 (99.27)	6715 (56.08)	10,419 (66.70)	2092 (75.77)
Marital status						
Others	596 (1.26)	156 (1.49)	71 (1.08)	188 (1.57)	151 (0.97)	30 (1.09)
Married or remarriage	46,788 (98.74)	10,313 (98.51)	6488 (98.92)	11,786 (98.43)	15,470 (99.03)	2731 (98.91)
Education background						
Illiterate/elementary	3136 (6.62)	696 (6.65)	375 (5.72)	763 (6.37)	1110 (7.11)	192 (6.95)
Middle school	31,000 (65.42)	6657 (63.59)	4096 (62.45)	7807 (65.20)	10,498 (67.20)	1942 (70.34)
High school or above	13,248 (27.96)	3116 (29.76)	2088 (31.83)	3404 (28.43)	4013 (25.69)	627 (22.71)
BMI, kg/m^2^	25.05 ± 3.28	24.31 ± 3.04	26.14 ± 3.01	23.88 ± 3.15	25.65 ± 3.23	26.86 ± 3.12
SBP, mm Hg	130.06 ± 18.43	127.70 ± 16.67	132.03 ± 16.92	125.90 ± 19.05	132.69 ± 18.78	137.59 ± 18.11
DBP, mm Hg	84.14 ± 10.50	83.70 ± 10.05	86.96 ± 10.24	81.07 ± 10.39	84.98 ± 10.33	87.70 ± 10.57
LDL-C, mmol/L	2.62 ± 0.73	2.59 ± 0.66	2.73 ± 0.71	2.43 ± 0.72	2.72 ± 0.75	2.72 ± 0.86
HDL-C, mmol/L	1.55 ± 0.42	1.61 ± 0.43	1.49 ± 0.40	1.67 ± 0.44	1.48 ± 0.38	1.36 ± 0.38
Hs-CRP, mg/L	1.00 (0.50–2.35)	0.97 (0.50–2.10)	1.20 (0.60–2.60)	0.93 (0.50–2.05)	1.00 (0.40–2.50)	1.51 (0.70–3.36)
eGFR, ml/min/1.73 m^3^	93.11 (76.59-104.54)	99.23 (86.11-108.22)	99.47 (85.25-108.11)	91.04 (74.39-104.02)	86.41 (71.31–99.61)	89.68 (74.06-102.16)
Hypertension	22,310 (47.08)	4471 (42.71)	3767 (57.43)	4149 (34.65)	8115 (51.95)	1808 (65.48)
Diabetes mellitus	4907 (10.36)	366 (3.50)	1103 (16.82)	318 (2.66)	1977 (12.66)	1143 (41.40)
Hyperlipidemia	16,730 (35.31)	2502 (23.90)	4378 (66.75)	1766 (14.75)	5742 (36.76)	2342 (84.82)
Medication usage						
Antihypertensive	5049 (10.66)	923 (8.82)	935 (14.26)	731 (6.10)	1924 (12.32)	536 (19.41)
Hypoglycemic	1390 (2.93)	82 (0.78)	278 (4.24)	1390 (2.93)	82 (0.78)	278 (4.24)
Hypolipidemic	368 (0.78)	49 (0.47)	93 (1.42)	368 (0.78)	49 (0.47)	93 (1.42)
TyG						
2006/07_TyG	8.63 ± 0.65	8.31 ± 0.42	9.20 ± 0.51	8.07 ± 0.36	8.83 ± 0.44	9.75 ± 0.52
2008/09_TyG	8.65 ± 0.63	8.38 ± 0.42	9.25 ± 0.49	8.09 ± 0.38	8.81 ± 0.39	9.77 ± 0.51
2010/11_TyG	8.68 ± 0.62	8.45 ± 0.42	9.29 ± 0.51	8.13 ± 0.36	8.81 ± 0.41	9.75 ± 0.52

Abbreviations: BMI, body mass index; SBP, systolic blood pressure; DBP, diastolic blood pressure; LDL-C, low-density lipoprotein cholesterol; HDL-C, high-density lipoprotein cholesterol; eGFR, estimated glomerular filtration rate; Hs-CRP, high sensitivity C-reactive protein; TyG, triglyceride-glucose

### Multi-trajectory groups of TyG levels and lifestyle scores and CVD

**Fig. 3:**
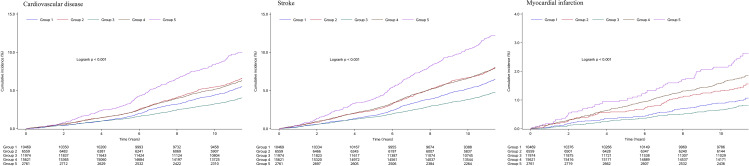
The Kaplan–Meier curves of cardiovascular disease, stroke, and myocardial infarction

During a median follow-up time of 10.98 (10.53–11.31) years, 3042 participants developed CVD events (2481 strokes, 616 myocardial infarctions, and 55 co-current stroke and myocardial infarctions). The highest incidence rate of CVD was observed in participants in group 5 with the highest TyG levels and moderate lifestyle scores (11.22 per 1000 person-years), while the lowest incidence rate was observed in participants in group 3 with the lowest TyG levels and the best lifestyle scores (4.09 per 1000 person‐years). The Kaplan-Meier curves also showed that participants in group 5 experienced higher risk of CVD, stroke, and myocardial infarction than those in other groups (*P* < 0.0001 for log‐rank test; Fig. 3).

After adjustment for potential confounders, participants in group 5, characterized by the highest TyG levels and moderate lifestyle scores, had the highest risk of CVD (HR = 1.76, 95% CI: 1.50–2.05). This risk was followed by group 2 with higher TyG levels and the poorest lifestyle scores (HR = 1.45, 95% CI: 1.26–1.66), and group 1 with lower TyG levels and poorer lifestyle scores (HR = 1.33, 95% CI: 1.17–1.50). Conversely, participants in group 4, with moderate TyG levels and better lifestyle scores, exhibited the lowest risk of CVD (HR = 1.32, 95% CI: 1.18–1.47). In the subtype analyses of CVD, similar results were yielded for stroke. However, participants in group 4 with moderate TyG levels and better lifestyle scores did not have the lowest risk of myocardial infarction (HR = 1.72, 95% CI: 1.34–2.21), but the second highest (Table 2).

**Table 2:** Incidence of CVD and its subtypes according to multi-trajectory of TyG levels and lifestyle scores

	Multi-trajectory of TyG levels and lifestyle scores, HR (95% CI)				
	Group 1	Group 2	Group 3	Group 4	Group 5
CVD					
N/n	616/10,469	482/6559	510/11,974	1124/15,621	310/2761
Incidence rate*	5.60(5.18–6.06)	7.03(6.43–7.69)	4.09(3.75–4.46)	7.02(6.62–7.44)	11.22(10.03–12.54)
Model 1	1.44(1.28–1.63)	1.98(1.74–2.25)	Reference	1.65(1.48–1.83)	2.72(2.36–3.13)
Model 2	1.44(1.27–1.62)	1.83(1.61–2.09)	Reference	1.53(1.38–1.71)	2.41(2.09–2.79)
Model 3	1.33(1.17–1.50)	1.45(1.26–1.66)	Reference	1.32(1.18–1.47)	1.76(1.50–2.05)
Stroke					
N/n	522/10,469	396/6559	428/11,974	882/15,621	253/2761
Incidence rate*	4.73(4.34–5.15)	5.74(5.20–6.33)	3.42(3.11–3.76)	5.47(5.12–5.84)	9.06(8.01–10.25)
Model 1	1.47(1.28–1.68)	1.95(1.69–2.24)	Reference	1.53(1.36–1.72)	2.63(2.25–3.07)
Model 2	1.46(1.28–1.67)	1.81(1.56–2.09)	Reference	1.43(1.27–1.61)	2.34(1.99–2.74)
Model 3	1.35(1.18–1.54)	1.44(1.24–1.68)	Reference	1.24(1.10–1.39)	1.73(1.46–2.06)
Myocardial infarction					
N/n	102/10,469	94/6559	89/11,974	266/15,621	65/2761
Incidence rate*	0.91(0.75–1.11)	1.34(1.09–1.64)	0.70(0.57–0.87)	1.63(1.44–1.83)	2.27(1.78–2.90)
Model 1	1.31(0.98–1.75)	2.10(1.55–2.84)	Reference	2.21(1.74–2.81)	3.15(2.29–4.34)
Model 2	1.30(0.97–1.74)	1.93(1.42–2.62)	Reference	2.05(1.61–2.62)	2.79(2.01–3.87)
Model 3	1.21(0.90–1.63)	1.49(1.09–2.04)	Reference	1.72(1.34–2.21)	1.86(1.31–2.64)

*Cases per 1000 person-years

Model 1 adjusted for age and sex

Model 2 included covariates in model 1 and marital status, education background, BMI

Model 3 included covariates in model 2 and LDL-C, HDL-C, Hs-CRP, eGFR, hypertension, diabetes mellitus, and use of antihypertensive, hypoglycemic, and hypolipidemic medications

**Fig. 4:**
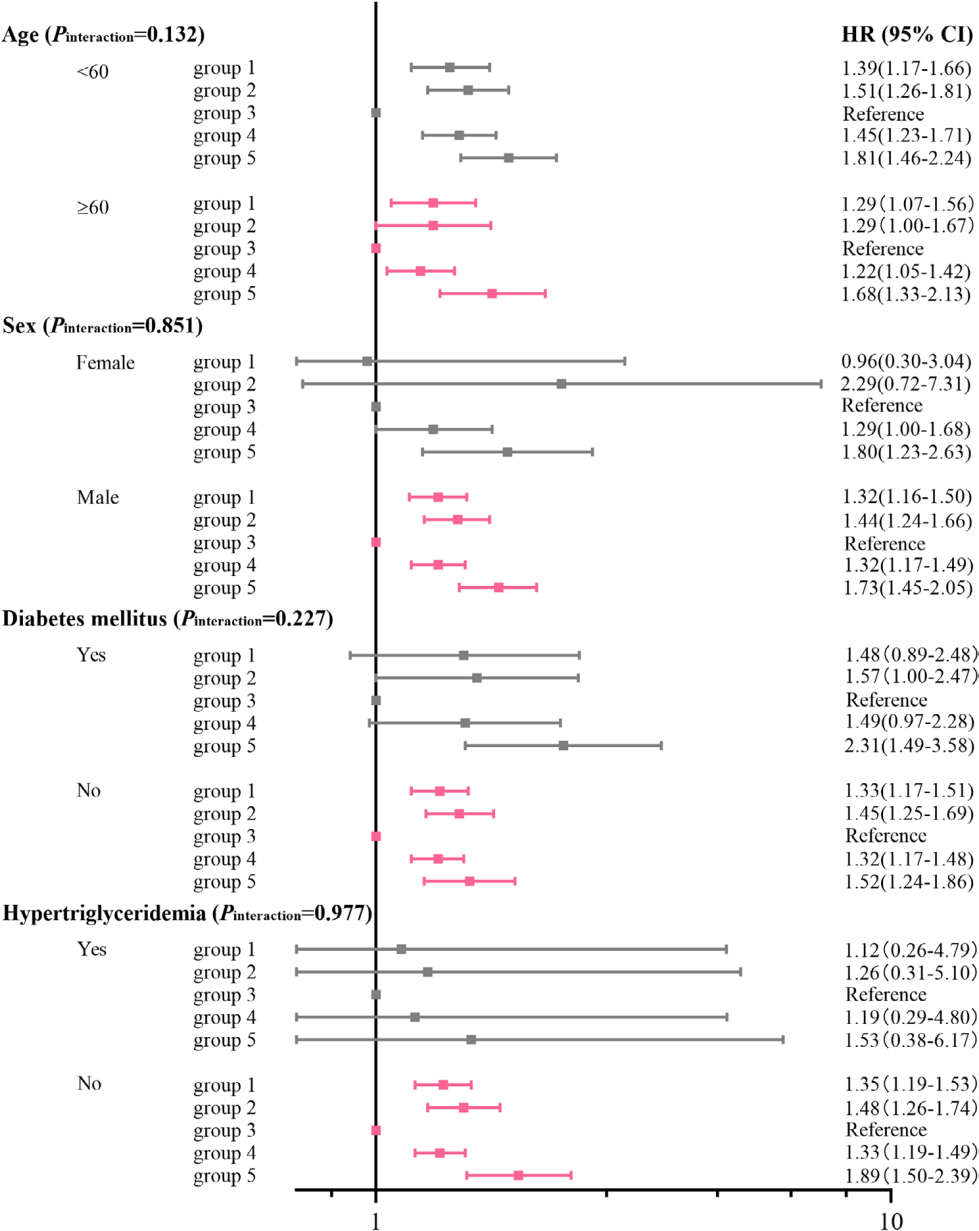
Stratification analyses by age, sex, diabetes mellitus and hypertriglyceridemia

Stratification analyses by age and sex showed the association between multi-trajectory groups of TyG levels and lifestyle scores and CVD were consistent across different subgroups. In contrast, we found associations consistent with the main results only in the non-diabetic and non-hypertriglyceridemia populations, which validated previous studies proposing that the use of the TyG index with CVD may be influenced by hypertriglyceridemia and diabetes [23]. There was no significant interaction between stratified variables and multi-trajectory groups in relation to the risk of CVD (all *P* values for interaction were > 0.05; Fig. 4). Additionally, adjustment for TyG levels and lifestyle scores at baseline did not change the associations materially (Supplementary Table 3). Sensitivity analysis with excluding medication usage (Supplementary Table 4), excluding Hs-CRP≥10 mg/L (Supplementary Table 5), excluding outcomes within the initial 2 years of follow-up (Supplementary Table 6), and treating non-CVD deaths as competing risk events (Supplementary Table 7), all generated similar findings to the primary analysis.

## Discussion

Through group-based multi-trajectory analysis of longitudinal data from 3 health surveys, we identified five distinct combined multi-trajectory groups of TyG levels and lifestyle scores. The findings indicated that participants in group 5, characterized by the highest TyG levels and moderate lifestyle scores, had the highest risk of CVD. This risk was followed by group 2 with higher TyG levels and the poorest lifestyle scores, and group 1 with lower TyG levels and poorer lifestyle scores. Conversely, participants in group 4, with moderate TyG levels and better lifestyle scores, exhibited the lowest risk of CVD. Notably, the application of the TyG index in CVD patients can be affected by increases in TG and glucose levels [23], and we found that the results remained robust in a non-diabetic and non-hypertriglyceridemic population, but the result of CVD in patients with hypertriglyceridemia or diabetes should be further verified.

Limited research has explored the relationship between TyG changes and CVD risk over time. A Chinese study identified three TyG trajectory classes, including low-stable, moderate-stable, and high-increasing, related to different carotid atherosclerosis progression risks, suggesting that TyG trajectories might serve as a valuable tool for identifying individuals at a higher risk [8]. A North American study supported this conclusion but with four TyG trajectories: low, moderate, high, and very high [9]. Earlier Kailuan Study results indicated that long-term TyG trajectories could predict CVD incidence in normal-weight adults [24]. Their study revealed five TyG trajectories. While our study also identified five distinct trajectories, the pattern development differed significantly, possibly due to our consideration of lifestyle influence.

In our study, lifestyle scores were derived from five factors: smoking, alcohol consumption, physical activity, sedentary behaviors, and salt intake. Previous research from our team revealed that these lifestyle trajectory patterns were associated with CVD and mortality risks [13]. Subsequently, a UK BioBank study established a lifestyle score encompassing smoking, physical activity, diet, body mass index, and sleep duration, highlighting an increased risk of ischemic heart disease with declining lifestyle trajectories [25]. Although no direct parallels exist, the trajectory of cardiovascular health scores has been widely studied since the American Heart Association proposed the 7 cardiovascular health metrics. Various studies have demonstrated the efficacy of cardiovascular health trajectories in predicting CVD development [15, 26, 27], indirectly supporting our perspective.

However, previous studies using trajectory modeling only investigated longitudinal changes in TyG levels and lifestyle scores separately, the present study provided new insights into identifying and monitoring joint developmental trajectories of both factors. Employing group-based multi-trajectory modeling, we uniquely defined trajectory groups based on two indicators, allowing us to capture their interrelationships and visualize their joint developmental patterns over time.

We found that different patterns of combined TyG levels and lifestyle scores were associated with varying risks of CVD. Interestingly, compared to the reference group with the lowest TyG levels and the best lifestyle scores, the pattern with the highest TyG levels and moderate lifestyle scores had the highest risk of CVD, whereas the pattern with moderate TyG levels and better lifestyle scores had the lowest risk of CVD. As we know, the TyG index serves as a reliable predictor of insulin resistance. Elevated TyG levels indicate the presence of insulin resistance (> 8.81 in men and > 8.73 in women) [28], which serves as both a pathogenic cause of metabolic abnormalities and a predictor of CVD. Simulated clinical trials using data from the National Health and Nutrition Examination Survey (NHANES) demonstrated that individuals with insulin resistance had a nearly 3-fold higher risk of coronary artery disease compared to those without [29]. Moreover, insulin resistance could remain latent for years before diabetes manifests, and the resulting chronic hyperglycemia leads to oxidative stress, initiating an inflammatory response and even causing irreversible damage to vascular cells that cannot be fully mitigated by an improved lifestyle [30]. The results from our stratified analysis suggested that the importance of the multi-trajectory of TyG levels and lifestyle scores might vary among different subgroups. In individuals without diabetes or hypertriglyceridemia, the multi-trajectory of TyG levels and lifestyle scores appeared to be more sensitive for assessing CVD risk. Both hypertriglyceridemia and diabetes significantly heighten the risks of incident CVD [31]. In cases where participants were already affected by diabetes or hypertriglyceridemia, these factors per se may have already exerted a substantial influence on CVD risk. In such scenarios, the TyG index may provide relatively less additional information for assessing CVD risk. Additionally, it is also possible that patients previously diagnosed with diabetes or hypertriglyceridemia were under treatment or had adopted healthier habits, so their analytical parameters might be well controlled [32]. Our findings underscored the potential clinical application of the TyG index for CVD risk assessment, especially in populations without those overt risk factors.

Our results emphasized the importance of assessing TyG trajectory in CVD progression and taking lifestyle changes into account may help to refine CVD risk stratification and enable the administration of more targeted therapeutics or prevention measures. The advantages of the TyG index lie in its simplicity for clinical application, utilizing routine lab tests, and eliminating the need for insulin measurements. Although various surrogate markers for insulin resistance exist, such as lipid accumulation product (LAP), visceral obesity index (VAI) [33–35], atherogenic index of plasma (AIP) [33], etc., the TyG index stands out for its predictive power for cardiometabolic diseases [34]. For example, a study involving Thai police officers demonstrated a strong association between these insulin-resistance surrogate markers and a high prevalence of metabolic syndrome and hypertension. Additionally, the TyG index appeared to outperform other indices in predictive performance [35]. Similarly, a comparable outcome indicated that the TyG index might exhibit superior discriminative performance compared to AIP for predicting poor cardiovascular outcomes in patients with chronic coronary syndrome [34]. [38]We need to take effective treatment to control TyG levels much earlier. However, we did not deny the benefits of improving lifestyle. Active lifestyle changes can enhance lipid metabolism, decrease inflammation, stabilize blood glucose, and reduce blood pressure, offering sustainable cardiovascular benefits [11]. While our study noted a progressive rise in CVD risk with increasing TyG levels, this trend was independent of lifestyle modifications. Although lifestyle remains vital for general well-being, addressing TyG levels could be a pivotal intervention strategy in certain contexts.

Our research utilized a population-based cohort study with comprehensive data collection and CVD event tracking through follow-ups, allowing us to make inclusive adjustments for risk factors. We were not the first study to use multi-trajectory modeling [36, 37], but our study was the first to investigate joint trajectories of TyG levels and lifestyle scores in relation to CVD. Multi-trajectory analysis enhances the accuracy of individual-specific probabilities by considering interrelationships among multiple metrics. Several limitations should also be acknowledged. Firstly, trajectory models are data-driven and the number of groups is not predetermined [7], requiring cautious interpretation of the results. Secondly, trajectory analyses mandate a minimum of three independent time points [38]. To ensure sufficient follow-up time to capture cardiovascular events, we analyzed TyG and lifestyle data from three health surveys, potentially missing longer trends in TyG levels and lifestyle scores. Future research could encompass more examination intervals. Thirdly, the study was conducted in the context of a northern Chinese population, which may limit the generalization of the findings. Fourthly, despite the large sample size of this study, females constituted a small proportion (approximately 24% of the total), especially in stratified analyses, which limited statistical power.

## Conclusion

In conclusion, our study identified five distinct multi-trajectory groups of TyG levels and lifestyle scores over a 4-year follow-up period. The findings indicated that individuals with elevated TyG levels, despite relatively healthier lifestyles, continued to face an increased risk of CVD. This observation underscores the value of monitoring longitudinal TyG levels as a predictor of CVD and the need for early implementation of effective interventions to maintain optimal TyG levels, thus reducing CVD risk.

### Electronic supplementary material

Below is the link to the electronic supplementary material.

Supplementary Material 1

## Data Availability

The datasets used and/or analyzed during the current study are not publicly available but are available from the corresponding author at reasonable request.

## List of abbreviations

BMI: Body mass index
CI: Confidence interval
CVD: Cardiovascular disease
DBP: Diastolic blood pressure
eGFR: Estimated glomerular filtration rate
HDL-C: High-density lipoprotein cholesterol
Hs-CRP: High sensitivity C-reactive protein
HR: Hazard ratio
LDL-C: Low-density lipoprotein cholesterol
SBP: Systolic blood pressure
TyG: Triglyceride-glucose
