# Supplementary material for: Multi-trajectories of triglyceride-glucose index and lifestyle with Cardiovascular Disease: a cohort study
Source: Cardiovasc Diabetol. 2023 Dec 13;22:341. doi: 10.1186/s12933-023-02076-z (PMC10720233; doi:10.1186/s12933-023-02076-z)
Supplement: Supplementary file 1 — Supplementary Material 1 [file 12933_2023_2076_MOESM1_ESM.docx]

**Supplementary materials**

**Supplementary Table 1.** Definitions of poor (0 Point), intermediate (1 Point), and ideal (2 Points) for each component of lifestyle scores

**Supplementary Table 2.** Parameters based on model-adequacy criteria for multi-trajectories in the best model of each group

**Supplementary Table 3.** HRs and 95% CIs for incidence of CVD after adjusting baseline TyG levels and lifestyle scores

**Supplementary Table 4.** HRs and 95% CIs for incidence of CVD after excluding medication usage

**Supplementary Table 5.** HRs and 95% CIs for incidence of CVD after excluding Hs-CRP≥10 mg/L

**Supplementary Table 6.** HRs and 95% CIs for incidence of CVD after excluding outcomes within the initial 2 years of follow-up

**Supplementary Table 7.** HRs and 95% CIs for incidence of CVD treating non-CVD deaths as competing risk events

**Supplementary Table 1.** Definitions of poor (0 Point), intermediate (1 Point), and ideal (2 Points) for each component of lifestyle scores

| Lifestyle score component | Poor (0 Point) | Intermediate (1 Point) | Ideal (2 Points) |
| --- | --- | --- | --- |
| Smoking | Current smoker | Past smoker | Never |
| Alcohol consumption | Current drinker | Past drinker | Never |
| Sedentary time, h/day | ≥8 | 4-7 | <4 |
| Physical activity (moderate or vigorous exercise) | No physical activity | physical activity (20+ minutes per time) 1-2 times per week, during leisure time | physical activity (20+ minutes per time) 3 times per week, during leisure time |
| Diet, based on daily salt intake (g/d) | ≥10 | 6-9 | <6 |

**Supplementary Table 2.** Parameters based on model-adequacy criteria for multi-trajectories in the best model of each group

| Variables | Number of groups | Order of trajectory | Proportion of individuals in groups (%) | BIC | 2∆BIC | AIC | Average posterior probability of assignment |
| --- | --- | --- | --- | --- | --- | --- | --- |
| Lifestyle scores | 2 | 1/1 | 38.06/61.94 | -424106.51 | - | -424053.91 | 0.92/0.95 |
| TyG levels | 2 | 2/1 |  |  |  |  |  |
| Lifestyle scores | 3 | 1/1/1 | 27.97/51.70/20.33 | -413516.08 | 21180.86 | -413445.95 | 0.87/0.92/0.87 |
| TyG levels | 3 | 1/1/1 |  |  |  |  |  |
| Lifestyle scores | 4 | 1/1/1/1 | 26.65/11.43/38.44/23.48 | -407094.89 | 12842.38 | -406998.46 | 0.86/0.86/0.88/0.84 |
| TyG levels | 4 | 1/2/1/1 |  |  |  |  |  |
| **Lifestyle scores** | **5** | **1/1/1/1/1** | **21.90/13.97/25.76/32.26/6.11** | **-403847.08** | **6495.62** | **-403715.59** | **0.85/0.84/0.85/0.83/0.85** |
| **TyG levels** | **5** | **1/2/2/2/2** |  |  |  |  |  |
| Lifestyle scores | 6 | 1/1/1/1/1/1 | 16.76/17.35/3.86/20.50/32.93/8.59 | -401672.5 | 4349.2 | -401527.8 | 0.82/0.82/0.85/0.83/0.82/0.83 |
| TyG levels | 6 | 1/1/2/1/2/1 |  |  |  |  |  |

Abbreviations: TyG, triglyceride-glucose; AIC, Akaike information criterion; BIC, Bayesian information criterion.

**Supplementary Table 3.** HRs and 95% CIs for incidence of CVD after adjusting baseline TyG levels and lifestyle scores

|  | Multi-trajectory of TyG levels and lifestyle scores, HR (95% CI) | | | | |
| --- | --- | --- | --- | --- | --- |
|  | Group 1 | Group 2 | Group 3 | Group 4 | Group 5 |
| CVD |  |  |  |  |  |
| N/n | 616/10469 | 482/6559 | 510/11974 | 1124/15621 | 310/2761 |
| Incidence rate* | 5.60(5.18-6.06) | 7.03(6.43-7.69) | 4.09(3.75-4.46) | 7.02(6.62-7.44) | 11.22(10.03-12.54) |
| Model 1 | 1.27(1.11-1.45) | 1.26(1.06-1.50) | Reference | 1.22(1.08-1.38) | 1.48(1.21-1.81) |
| Stroke |  |  |  |  |  |
| N/n | 522/10469 | 396/6559 | 428/11974 | 882/15621 | 253/2761 |
| Incidence rate* | 4.73(4.34-5.15) | 5.74(5.20-6.33) | 3.42(3.11-3.76) | 5.47(5.12-5.84) | 9.06(8.01-10.25) |
| Model 1 | 1.29(1.11-1.50) | 1.31(1.08-1.58) | Reference | 1.19(1.04-1.36) | 1.58(1.27-1.98) |
| Myocardial infarction |  |  |  |  |  |
| N/n | 102/10469 | 94/6559 | 89/11974 | 266/15621 | 65/2761 |
| Incidence rate* | 0.91(0.75-1.11) | 1.34(1.09-1.64) | 0.70(0.57-0.87) | 1.63(1.44-1.83) | 2.27(1.78-2.90) |
| Model 1 | 1.12(0.81-1.55) | 1.05(0.71-1.55) | Reference | 1.36(1.03-1.80) | 1.11(0.71-1.74) |

*Cases per 1000 person-years.

Model 1 adjusted for age, sex, marital status, education background, body mass index, LDL-C, HDL-C, Hs-CRP, eGFR, hypertension, diabetes mellitus, use of antihypertensive, hypoglycemic, and hypolipidemic medications, TyG levels and lifestyle scores at baseline

**Supplementary Table 4.** HRs and 95% CIs for incidence of CVD after excluding medication usage

|  | Multi-trajectory of TyG levels and lifestyle scores, HR (95% CI) | | | | |
| --- | --- | --- | --- | --- | --- |
|  | Group 1 | Group 2 | Group 3 | Group 4 | Group 5 |
| Excluding antihypertensive usage | | |  |  |  |
| N/n | 512/9546 | 370/5624 | 443/11243 | 903/13697 | 217/2225 |
| Incidence rate* | 5.08(4.66-5.54) | 6.24(5.64-6.91) | 3.77(3.44-4.14) | 6.38(5.98-6.81) | 9.63(8.43-11.00) |
| Model | 1.35(1.18-1.54) | 1.49(1.28-1.73) | Reference | 1.33(1.18-1.50) | 1.71(1.43-2.04) |
| Excluding hypoglycemic usage | | |  |  |  |
| N/n | 606/10387 | 452/6281 | 504/11899 | 1063/15048 | 246/2379 |
| Incidence rate* | 5.55(5.13-6.01) | 6.87(6.27-7.53) | 4.06(3.72-4.43) | 6.87(6.47-7.30) | 10.27(9.07-11.64) |
| Model | 1.33(1.18-1.51) | 1.47(1.28-1.69) | Reference | 1.32(1.18-1.48) | 1.70(1.44-2.00) |
| Excluding hypolipidemic usage | | |  |  |  |
| N/n | 609/10420 | 472/6466 | 508/11949 | 1117/15494 | 303/2687 |
| Incidence rate* | 5.56(5.14-6.02) | 6.98(6.38-7.64) | 4.08(3.74-4.45) | 7.03(6.63-7.46) | 11.28(10.08-12.63) |
| Model | 1.32(1.17-1.49) | 1.44(1.26-1.66) | Reference | 1.32(1.18-1.48) | 1.76(1.51-2.06) |
| Excluding all medication usage | | |  |  |  |
| N/n | 502/9460 | 347/5406 | 439/11177 | 869/13294 | 195/2002 |
| Incidence rate* | 5.02(4.60-5.48) | 6.08(5.47-6.76) | 3.76(3.42-4.13) | 6.32(5.91-6.75) | 9.63(8.37-11.08) |
| Model | 1.35(1.18-1.54) | 1.49(1.27-1.74) | Reference | 1.33(1.18-1.50) | 1.75(1.46-2.10) |

*Cases per 1000 person-years.

Model adjusted for age, sex, marital status, education background, body mass index, LDL-C, HDL-C, Hs-CRP, eGFR, hypertension, diabetes mellitus, and use of antihypertensive, hypoglycemic, and hypolipidemic medications

**Supplementary Table 5.** HRs and 95% CIs for incidence of CVD after excluding Hs-CRP≥10 mg/L

|  | Multi-trajectory of TyG levels and lifestyle scores, HR (95% CI) | | | | |
| --- | --- | --- | --- | --- | --- |
|  | Group 1 | Group 2 | Group 3 | Group 4 | Group 5 |
| N/n | 604/10383 | 471/6509 | 486/11867 | 1079/15423 | 301/2725 |
| Incidence rate* | 5.50(5.08-5.95) | 6.88(6.28-7.53) | 3.90(3.57-4.26) | 6.75(6.35-7.16) | 10.90(9.74-12.21) |
| Model | 1.35(1.19-1.53) | 1.46(1.27-1.68) | Reference | 1.33(1.19-1.49) | 1.79(1.53-2.09) |

Abbreviation: Hs-CPR, high sensitivity C-reactive protein.

*Cases per 1000 person-years.

Model adjusted for age, sex, marital status, education background, body mass index, LDL-C, HDL-C, Hs-CRP, eGFR, hypertension, diabetes mellitus, and use of antihypertensive, hypoglycemic, and hypolipidemic medications

**Supplemental Table 6.** HRs and 95% CIs for incidence of CVD after excluding outcomes within the initial 2 years of follow-up

|  | Multi-trajectory of TyG levels and lifestyle scores, HR (95% CI) | | | | |
| --- | --- | --- | --- | --- | --- |
|  | Group 1 | Group 2 | Group 3 | Group 4 | Group 5 |
| N/n | 604/10383 | 471/6509 | 486/11867 | 1079/15423 | 301/2725 |
| Incidence rate* | 5.50(5.08-5.95) | 6.88(6.28-7.53) | 3.90(3.57-4.26) | 6.75(6.35-7.16) | 10.90(9.74-12.21) |
| Model | 1.35(1.19-1.53) | 1.46(1.27-1.68) | Reference | 1.33(1.19-1.49) | 1.79(1.53-2.09) |

*Cases per 1000 person-years.

Model adjusted for age, sex, marital status, education background, body mass index, LDL-C, HDL-C, Hs-CRP, eGFR, hypertension, diabetes mellitus, and use of antihypertensive, hypoglycemic, and hypolipidemic medications

**Supplemental Table 7.** HRs and 95% CIs for incidence of CVD treating non-CVD deaths as competing risk events

|  | Multi-trajectory of TyG levels and lifestyle scores, HR (95% CI) | | | | |
| --- | --- | --- | --- | --- | --- |
|  | Group 1 | Group 2 | Group 3 | Group 4 | Group 5 |
| N/n | 616/10469 | 482/6559 | 510/11974 | 1124/15621 | 310/2761 |
| Incidence rate* | 5.60(5.18-6.06) | 7.03(6.43-7.69) | 4.09(3.75-4.46) | 7.02(6.62-7.44) | 11.22(10.03-12.54) |
| Model | 1.35(1.18-1.55) | 1.44(1.24-1.68) | Reference | 1.24(1.10-1.40) | 1.74(1.47-2.07) |

*Cases per 1000 person-years.

Model adjusted for age, sex, marital status, education background, body mass index, LDL-C, HDL-C, Hs-CRP, eGFR, hypertension, diabetes mellitus, and use of antihypertensive, hypoglycemic, and hypolipidemic medications
